# High order mode structure of intense light fields generated via a laser-driven relativistic plasma aperture

**DOI:** 10.1038/s41598-019-57119-x

**Published:** 2020-01-09

**Authors:** M. J. Duff, R. Wilson, M. King, B. Gonzalez-Izquierdo, A. Higginson, S. D. R. Williamson, Z. E. Davidson, R. Capdessus, N. Booth, S. Hawkes, D. Neely, R. J. Gray, P. McKenna

**Affiliations:** 1 SUPA Department of Physics, University of Strathclyde, Glasgow, G4 0NG UK; 2 Central Laser Facility, STFC Rutherford Appleton Laboratory, Oxfordshire, OX11 0QX UK

**Keywords:** Laser-produced plasmas, Plasma-based accelerators

## Abstract

The spatio-temporal and polarisation properties of intense light is important in wide-ranging topics at the forefront of extreme light-matter interactions, including ultrafast laser-driven particle acceleration, attosecond pulse generation, plasma photonics, high-field physics and laboratory astrophysics. Here, we experimentally demonstrate modifications to the polarisation and temporal properties of intense light measured at the rear of an ultrathin target foil irradiated by a relativistically intense laser pulse. The changes are shown to result from a superposition of coherent radiation, generated by a directly accelerated bipolar electron distribution, and the light transmitted due to the onset of relativistic self-induced transparency. Simulations show that the generated light has a high-order transverse electromagnetic mode structure in both the first and second laser harmonics that can evolve on intra-pulse time-scales. The mode structure and polarisation state vary with the interaction parameters, opening up the possibility of developing this approach to achieve dynamic control of structured light fields at ultrahigh intensities.

## Introduction

Control of the spatio-temporal and polarisation properties of high power laser pulses is important to the development of compact laser-plasma-based particle accelerators and radiation sources, which have wide-ranging potential applications^1–4^. Changing the spatial modes of focused laser light enables tailoring of transverse focusing forces within the plasma^5^, resulting in enhanced electron and positron acceleration^6,7^, attosecond electron bunches^8^, stabilised ion acceleration^9^, and the possibility to generate X-rays with orbital angular momentum^10,11^. Moreover, it has been shown numerically that the focusing forces can be controlled by changing the relative intensity ratio of two laser modes used in combination^12^. Similarly, collective particle dynamics and field generation in plasma can be tailored by varying the drive laser polarisation^13,14^, enabling new degrees of control in charged particle acceleration^13,15,16^. An ability to dynamically vary these laser properties at high power could have a transformational effect on topics at the forefront of ultraintense light-matter interactions, plasma photonics and radiation generation.

As a medium, plasma can withstand extremely high energy densities and its optical properties can be varied on the ultrafast timescale of a high power laser pulse, enabling the possibility for dynamic manipulation of intense light. This motivates investigation of relativistic plasma optical and photonics phenomena. The use of plasma to amplify^17–19^, compress^20^ and condition^21–24^ laser pulses has been explored. Recently, it has been shown that magnetic splitting^25^, birefringence^26,27^ and density grating structures^28^ produced in low-density plasma could be used to control the polarisation of intense laser light.

**Figure 1:**
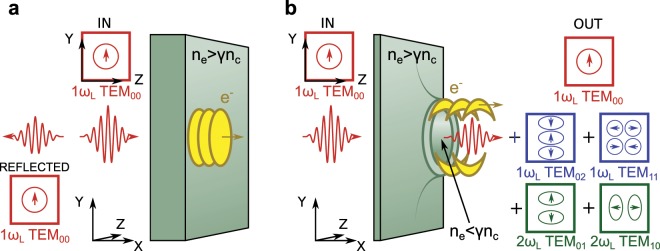
(**a**) In the absence of relativistic transparency, an incoming, linearly polarised laser pulse, in the form of a fundamental TEM_00_ (or Gaussian mode, indicated by the red box in which the arrow represents the polarisation direction), produces a current of energetic electrons bunched at *ω*_*L*_ and 2*ω*_*L*_ and reflects from the overdense plasma. (**b**) In an ultrathin foil, relativistic self-induced transparency results in a plasma aperture in which the linearly polarised (along the *Y* axis) laser light is transmitted and produces a bipolar distribution of dense electron bunches at the aperture edge. The deceleration of these bunches in the electrostatic sheath field produces coherent light emission at *ω*_*L*_ (blue boxes) and 2*ω*_*L*_ (green boxes) in higher order TEM modes. The arrow heads show the local polarisation direction of these modes.

Laser light of wavelength *λ*_*L*_ and angular frequency *ω*_*L*_ cannot propagate in a plasma with density *n*_*e*_ above a critical value, ${n}_{c}={m}_{e}{\varepsilon }_{0}{\omega }_{L}^{2}/{e}^{2}$, where *m*_*e*_ is the electron rest mass and *ε*_0_ is the vacuum permittivity^29^, and thickness greater than the skin depth $(d\gg {\ell }_{s})$. The skin depth is the distance over which the laser field decays in magnitude to 1/*e* of its peak value, and is defined as ${\ell }_{s}=c\sqrt{{\gamma }_{e}}/{\omega }_{p,e}$, where ${\omega }_{p,e}=\sqrt{{n}_{e}{e}^{2}/{\varepsilon }_{0}{m}_{e}}$ is the plasma frequency and *γ*_*e*_ is the electron Lorentz factor. Bunches of energetic electrons are injected into the target via mechanisms such as resonance absorption (at *ω*_*L*_) and **j** × **B** heating (at 2*ω*_*L*_)^30,31^, as illustrated in Fig. 1(a). These electrons transit the target and emerge at the rear solid-vacuum interface, where they generate transition radiation as a result of the change in the dielectric constant at the boundary. Typically, this will be in the form of a cylindrically symmetric intensity distribution, and if the coherence structure of the fast electron bunches is retained in the transport across the target, it will take the form of coherent transition radiation at *ω*_*L*_ and/or 2*ω*_*L*_^32,33^ (depending on the dominant absorption mechanism). The energetic electrons also produce an electric sheath field resulting in ion acceleration^34^. At relativistic laser intensities, the mass of the oscillating electrons increases by *γ*_*e*_, such that a region of the plasma, for which ${n}_{e} < {\gamma }_{e}{n}_{c}$, undergoes so-called relativistic self-induced transparency (RSIT)^35,36^. The intensity threshold at which this occurs in ultrathin foils $(d \sim {\ell }_{s})$ is reduced by plasma expansion, which decreases *n*_*e*_. A relativistically-transparent plasma aperture is produced at the most intense region of a typical Gaussian laser focus, as discussed in reference^14^ and illustrated in Fig. 1(b). In this case, the laser pulse propagating through the aperture can directly accelerate electrons (See reference^37^ for a discussion of direct electron acceleration in sub-critical density plasma). This case of an ultrathin foil undergoing RSIT is of particular importance for the acceleration of ions to high energies^38^ and for the generation of bright attosecond pulses of XUV radiation^39^.

Here, the effects of the self-generated relativistic plasma aperture in an expanding ultrathin foil on the intense light field at the target rear is explored experimentally and numerically via 3D Particle-In-Cell (PIC) code simulations. It is shown that measured changes to the polarisation and temporal properties of the light result from the generation of first and second harmonic radiation in high-order transverse electromagnetic modes. These are produced by dense, coherent bunches of electrons that are directly accelerated from the edges of the aperture in a bipolar distribution, as illustrated in Fig. 1(b). It is shown that the polarisation and degree of conversion to a given mode varies with the energy of the light detected at the rear of the target, making this both a diagnostic of the intra-pulse time at which RSIT occurs and a potentially tunable approach to producing high-order modes of relativistically intense laser light.

## Results

### Experimental and numerical investigation of polarisation

We begin with results from an experiment performed to explore changes to the polarisation of light transmitted through ultrathin foils expanding to near critical densities and undergoing RSIT. This was motivated in part by the theoretical and numerical work reported in Stark *et al*.^40^ that predicts that anisotropic heating of near critical density plasma by linearly-polarised light can induce a phase separation of two orthogonal modes and thus act like a waveplate. The parameters of our experiment, presented in the Methods Section, differ from the numerical work explored in reference^40^ in several ways, particularly with respect to target thickness and density evolution. In our experiment, aluminium foils, with thickness *d*, varied in the range 16–60 nm, were irradiated at close to normal incidence with linearly polarised light with a spot size of (3.9 ± 0.7) *μ*m (FWHM) and peak intensity equal to (2.8 ± 0.4) × 10^20^ Wcm^−2^.

Light collected at the rear of the plasma was collimated and directed to a Stokes polarimeter diagnostic (see Methods section and the Supplementary Information file for details). The Stokes parameters (S_1_, S_2_ and S_3_) were measured to provide a time-integrated measurement of the polarisation state of the light. The temporal-frequency profile of the light was measured using the frequency-resolved optical gating (FROG) technique^41^, using a sub-sample of the collimated beam (see Supplementary Information file for more details). Both sets of measurements were made as a function of target thickness.

**Figure 2:**
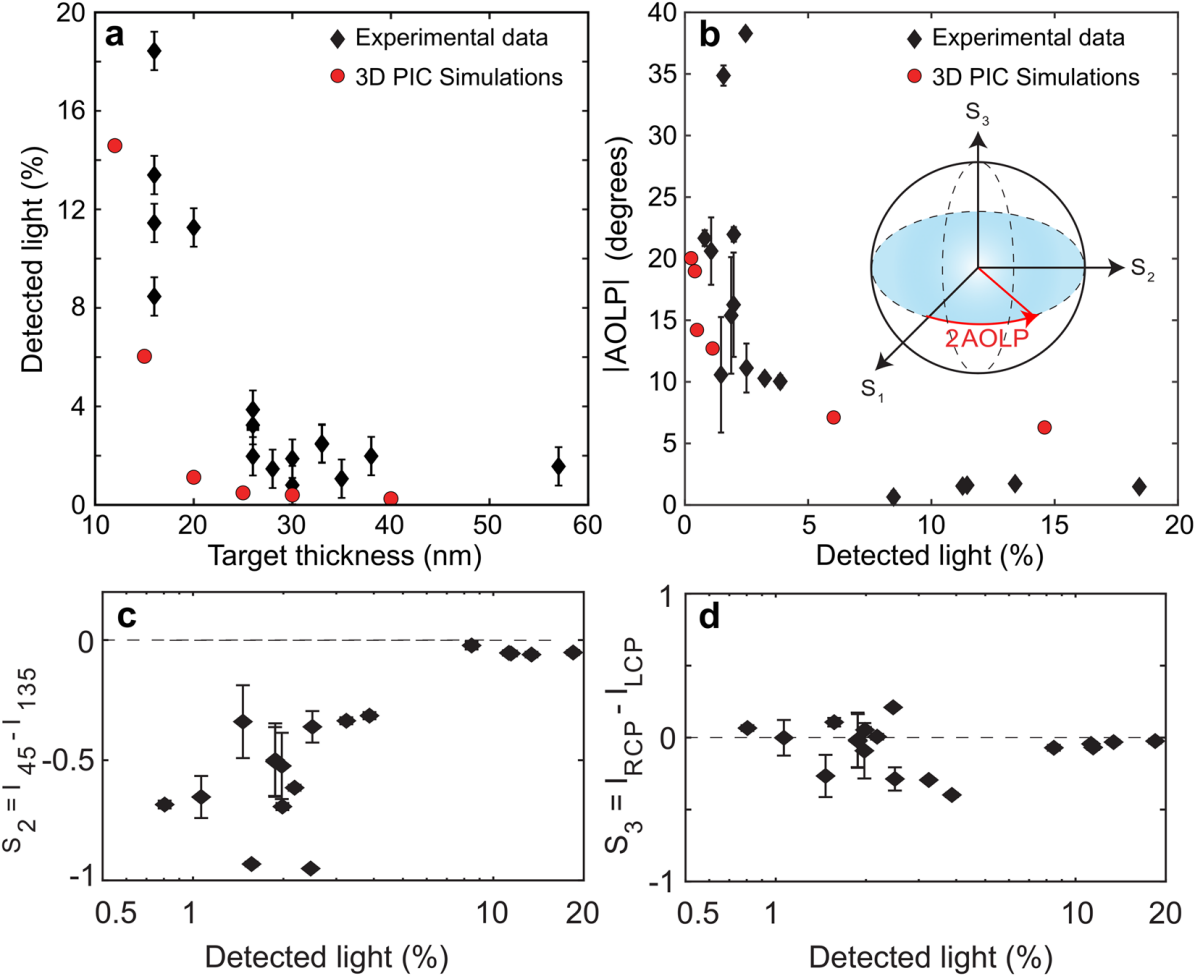
(**a**) Detected *ω*_*L*_ light level (energy as a percentage of the laser energy incident on the target) as a function of target thickness: Experiment - black; Simulations - red. The error bars correspond to the standard deviation in the transmission for a given target thickness, and thus account for fluctuations in the laser parameters. (**b**) Magnitude of the change in the angle of linear polarisation (AOLP) as a function of detected *ω*_*L*_ light level (energy as a percentage of the laser energy incident on the target): Experiment - black; Simulations - red. The error bars indicate the confidence in the polarisation state determined by the analysis code (see the Methods for more details). The inset illustrates the AOLP in the Poincaré sphere, where the Stokes parameters form an orthonormal basis. (**c**) Measured *S*_2_ parameter as a function of the detected light energy. (**d**) Same for the *S*_3_ parameter.

Figure 2a presents measurements of the energy of the light detected downstream of the target rear, normalised to the laser energy incident on the target. The energy of the detected light increases non-linearly with decreasing *d*, consistent with previous results in the same parameter regime^14,42^. Figure 2b shows the measured magnitude of the angle of linear polarisation (AOLP) as a function of the total energy of the light detected downstream of the target rear, again normalised to the laser energy on target. AOLP is proportional to the arctan of the ratio of the magnitudes of the measured S_2_ and S_1_ Stokes parameters, as shown in the inset. Both of these Stokes parameters vary with the level of light detected, whereas the *S*_3_ parameter does not (the measured values are scattered randomly around the reference value), as shown in Fig. 2c (for the *S*_2_ case) and Fig. 2d, respectively. Thus, AOLP is an appropriate figure of merit for quantifying the magnitude of the effective polarisation shift with respect to the incident laser light. A strong inverse correlation is observed between the AOLP value and the energy of light detected. The largest change in AOLP is measured when RSIT occurs late in the interaction, such that the transmitted light energy is low, and vice versa.

**Figure 3:**
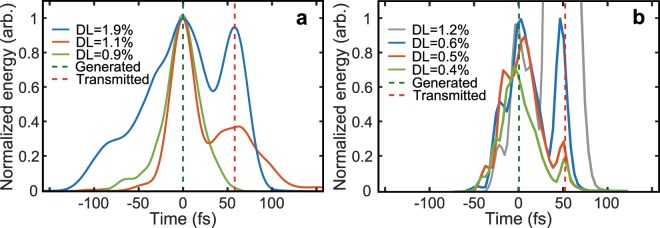
(**a**) Temporal-intensity profile of the light pulses measured using the FROG diagnostic for stated levels of detected light (‘DL’); (**b**) Same from the 3D PIC simulations. The dashed lines label the light generated in the plasma and transmitted laser light.

The temporal-intensity profile of the light, as measured using the FROG diagnostic, is shown in Fig. 3a. A single peak is observed in the case of very low detected light, consistent with the emission of transition radiation. The signal varies following the laser pulse profile. A second peak, 55 fs later, corresponds to transmitted laser light and grows in magnitude rapidly with increased light detection. The temporal separation between the two peaks is explained by the fact that the target becomes relativistically transparent late in the interaction, on the falling side of the laser pulse, but the intensity of the transmitted light is equivalent to, or greater than, the transition radiation (depending on the precise time at which RSIT occurs).

Together, these two sets of measurements demonstrate that the light detected downstream of the target has two distinctive components (light generated in the plasma early in the interaction and laser light transmitted through it) and that the time-integrated polarisation state is strongly dependant on the level of detected light. The relative intensity of the two light sources and the effective polarisation state varies with target thickness (or other parameters upon which the onset of RSIT depends, e.g. pulse intensity).

The underlying physics was investigated via 3D simulations performed using the fully relativistic particle-in-cell (PIC) code, EPOCH^43^ (see Methods section). Figure 2a (red circles) shows the total energy of light (normalised to the incident laser energy) downstream of the target rear as a function of *d*. The simulation results follow a very similar scaling to the experimental data. The differences in the detected light energy indicate that the degree of pre-expansion used in setting up the simulation may not precisely match that in the experiment. The simulations show that an *E*_*Z*_ field is generated and that the ratio |*E*_*Z*_|/*|E*_*Y*_|, which produces the effective polarisation change, increases with *d* (and thus a decreasing detected light energy). The observed scaling compares well to that measured in experiment, as shown in Fig. 2b, and continues to reduce towards zero for decreasing *d*, and thus detected light energies higher than those plotted. Moreover, as shown in Fig. 3b, the simulations also show two distinct peaks in the magnitude of the light downstream from the target rear; one induced by light generated in the plasma and the second by light transmitted through it. Both the strong correlation in the level of the second peak to the total light detected and the temporal separation of the peaks are in good agreement with the experimental results in Fig. 3a.

### Spatial mode generation and correlation with polarisation state

**Figure 4:**
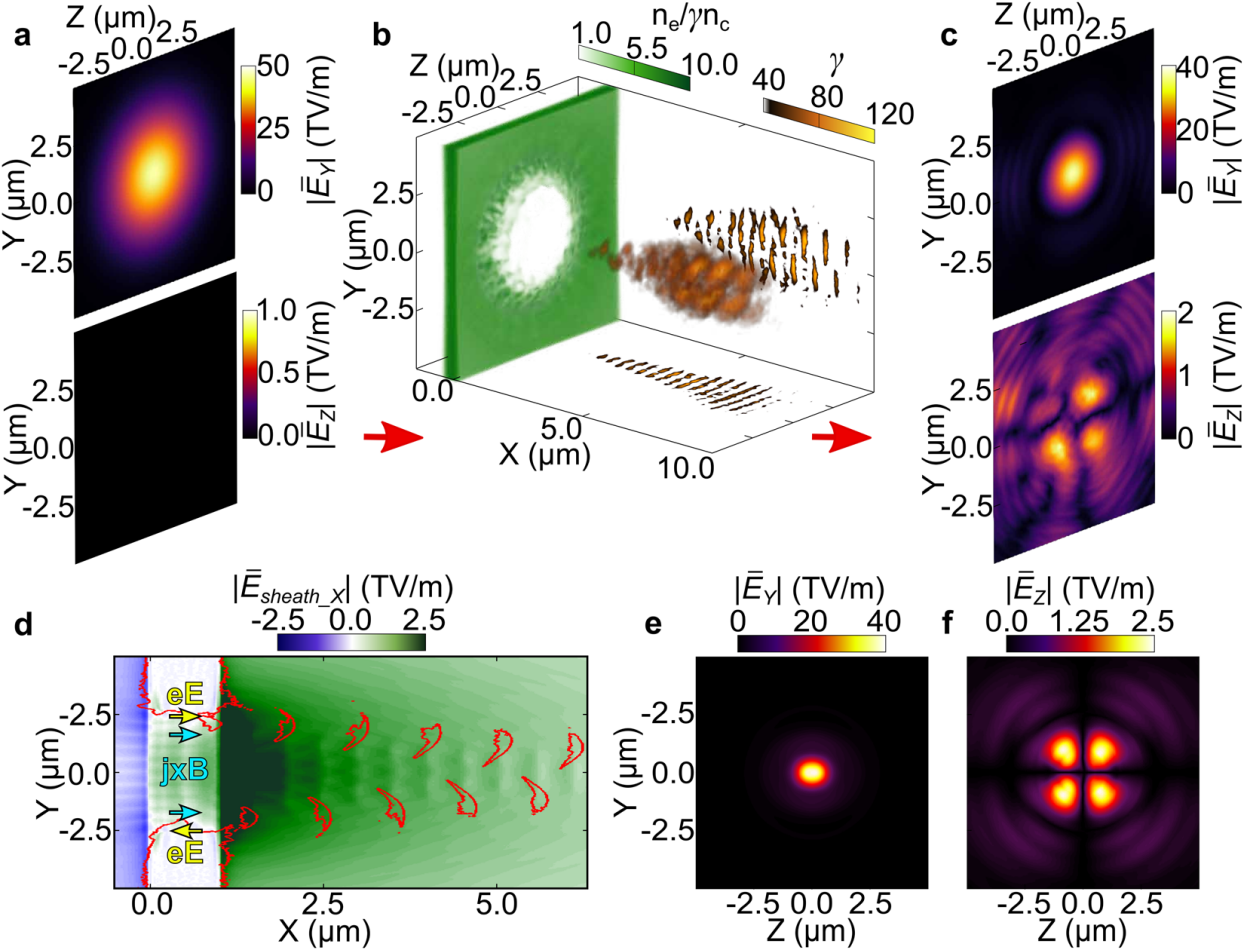
(**a**–**c**) Interaction of a Gaussian pulse with peak intensity equal to 6 × 10^20^ Wcm^−2^ with a *d* = 10 nm target. (**a**) The $|{\bar{E}}_{Y}|$ and $|{\bar{E}}_{Z}|$ components of the input pulse; (**b**) The relativistic plasma aperture and bipolar distribution of fast electrons accelerated in bunches from the edge of the aperture (at *t* = 40 fs). The bunch structures in the *X*-*Y* and *X*-*Z* planes are also shown; (**c**) The $|{\bar{E}}_{Y}|$ and $|{\bar{E}}_{Z}|$ components of the output light, at *X* = 10 *μ*m and *t* = 40 fs. (**d**–**f**) 3D PIC simulation results for the case of a target with a preformed, 5 *μ*m-diameter aperture: (**d**) Linearly polarised (*Y*-axis) laser light propagates in the + *X* direction. The red lines are *n*_*e*_ = *n*_*c*_ electron density contours and the colourmap indicates the magnitude of the spatially averaged longitudinal (sheath) electric field; (**e**) The transmitted $|{\bar{E}}_{Y}|$ field in the *Y*-*Z* plane; (**f**) The self-generated $|{\bar{E}}_{Z}|$ field in the same plane.

3D PIC simulation results further reveal that the changes in AOLP are produced by a superposition of TEM modes of the light generated during the laser-plasma interaction and the transmitted laser light. Figure 4a–c demonstrates the mode generation for *d* = 10 nm. Figure 4a shows the spatial distributions (*Y*-*Z* plane) of the $|{\bar{E}}_{Y}|$ and $|{\bar{E}}_{Z}|$ field components for the input laser pulse, where the bar indicates the fields are averaged over one laser period. The target electron density distribution is shown in Fig. 4b, 40 fs after interaction with the peak of the pulse. A relativistic aperture^14^ is formed at the most intense region of the laser focus. The spatial distributions of the $|{\bar{E}}_{Y}|$ and $|{\bar{E}}_{Z}|$ fields, 10 *μ*m behind the target and at *t* = 40 fs, are shown in Fig. 4c. At this distance the far-field distribution of the light is sampled, minimising the influence of near-field diffraction effects^14^. The measurements of the fields are filtered such that only *ω*_*L*_ light is present. Both the structure of the output fields and the ratio of the magnitude of $|{\bar{E}}_{Y}|$ and $|{\bar{E}}_{Z}|$ are of interest. A TEM_11_ mode is observed in $|{\bar{E}}_{Z}|$, as characterised by the four lobes. Only the fundamental TEM_00_ is observed in $|{\bar{E}}_{Y}|$ (the peak of which is ~20 times greater than the $|{\bar{E}}_{Z}|$ signal) because of high transmission due to the early onset of RSIT. The smallest effective polarisation shift is observed for this foil thickness, as seen in Fig. 2b.

The mode generation is explained by the production of dense electron bunches, shown in orange in Fig. 4b. The bunches are formed by the acceleration of electrons from the edges of the aperture. This occurs at two polar positions around the aperture (*Y*-*Z* plane) in the case of linearly polarised light. The overall bunch frequency is 2*ω*_*L*_, with consecutive bunches at each pole separated by *λ*_*L*_. The electron bunches are decelerated in the sheath field at the target rear side resulting in the emission of *ω*_*L*_ radiation in the polarisation plane, with the radiated *E*_*Y*_ field taking the form of a TEM_02_ mode. Later it will be shown that 2*ω*_*L*_ light is also generated.

The process is demonstrated by the 3D PIC simulation results shown in Fig. 4d–f, in which a simplified case of the laser interacting with a predefined 5 *μ*m diameter aperture is modelled. Electron bunches are formed at the edges of the aperture when the **j** × **B** force (blue arrow) and the force due to the focused laser *E*_*X*_ field (yellow arrow) are aligned in the same direction. The red contours correspond to *n*_*e*_ = *n*_*c*_ and show the propagation of the bunches through the longitudinal sheath field. The distributions of the $|{\bar{E}}_{Y}|$ and $|{\bar{E}}_{Z}|$ fields sampled 10 *μ*m behind the target are shown in Fig. 4e,f, respectively. Although the transmitted laser field still dominates, there is a clear TEM_11_ structure produced in the $|{\bar{E}}_{Z}|$ field. This has a higher signal-to-noise ratio than the results in Fig. 4c because the aperture is predefined.

**Figure 5:**
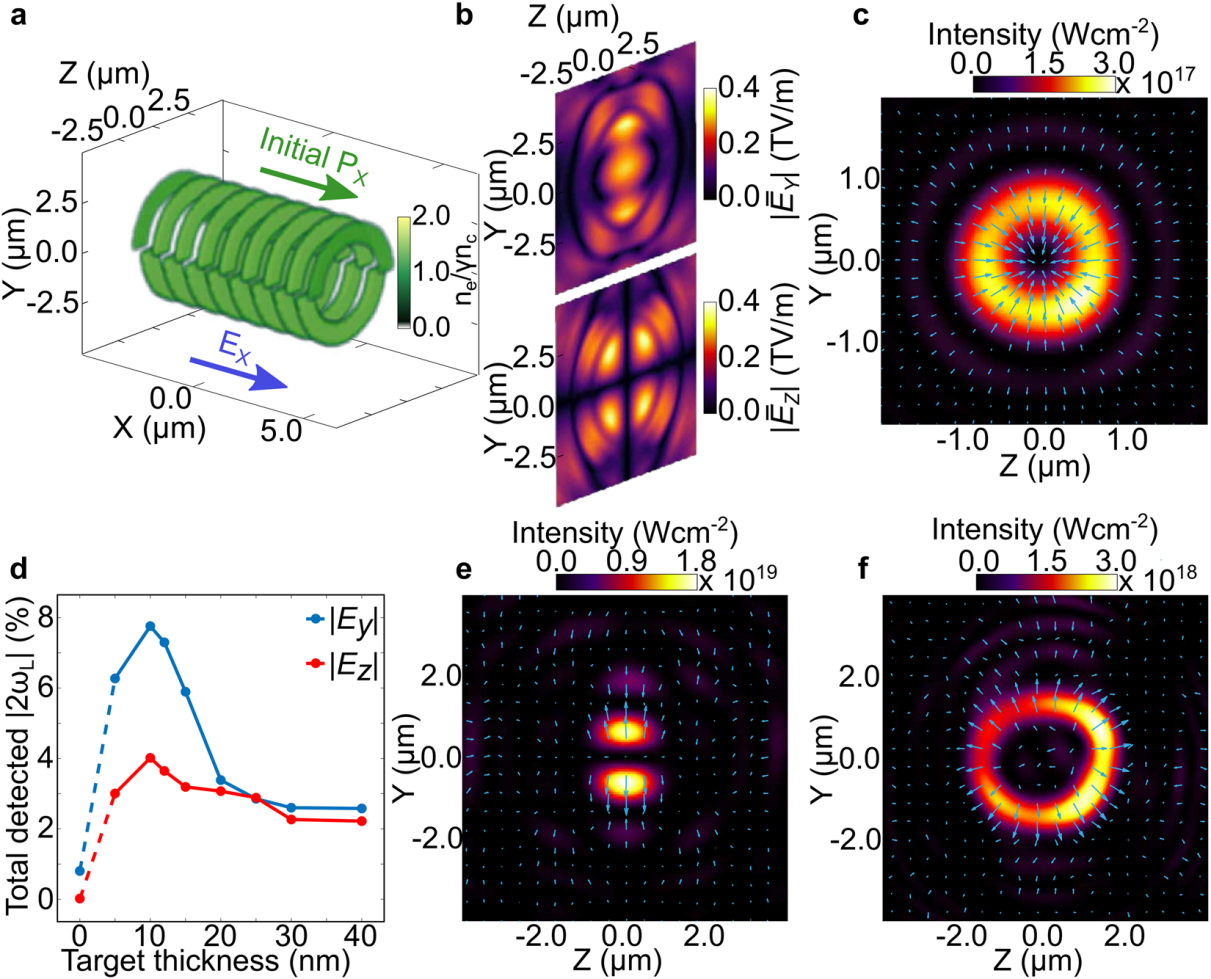
(**a**–**c)** Simulation results for a simplified case of preformed electron bunches propagating in a strong electrostatic field (i.e. no laser or target); (**a**) The initial electron density, momentum and *E*_*X*_ field directions defined in the simulation; (**b**) The $|{\bar{E}}_{Y}|$ and $|{\bar{E}}_{Z}|$ fields at *X* = 10 *μ*m; (**c**) Spatial intensity distribution and electric field vectors of the 2*ω*_*L*_ light at X = 10 *μ*m. (**d**–**f**) 2*ω*_*L*_ results from the full laser-foil simulations (no preformed aperture): (**d**) Total detected 2*ω*_*L*_ light, separated into the $|{\bar{E}}_{Y}|$ (blue) and $|{\bar{E}}_{Z}|$ (red) components, as a function of *d*. (**e**) Spatial intensity distribution and electric field vectors of the 2*ω*_*L*_ light for *d* = 5 nm; (**f**) Same for *d* = 20 nm.

To further explore the source of the TEM_02_ mode radiation, Fig. 5a–c shows results from a 3D PIC simulation in which predefined bunched electrons propagate in an electrostatic field, with no target or laser pulse. Bunches of electrons with *n*_*e*_ = *n*_*c*_, separated by *λ*_*L*_/2 and alternately mirrored about the *Z* = 0 plane, are initialised with a relativistic drift velocity in the +*X* direction and decelerated by a longitudinal 10 TVm^−1^ electric field (to replicate the effect of the sheath field at the target rear). The generated electromagnetic radiation propagates in the +*X* direction while the electrons decelerate and reverse direction. Figure 5b shows the spatial distributions of the generated $|{\bar{E}}_{Y}|$ and $|{\bar{E}}_{Z}|$ fields. As there is no input laser pulse, it is possible to clearly observe the TEM_02_ mode in *E*_*Y*_. This mode is present in all of the simulations presented above, but is masked by the stronger fundamental TEM_00_ mode. The TEM_11_ structure is also clearly observed in the *E*_*Z*_ field in Fig. 5b, confirming that the mechanism for generating the TEM_11_ is related to the decelerating electron bunches.

These simplified simulations show that the TEM_02_ mode is produced by the deceleration of the bipolar electron distribution. It is also possible to demonstrate analytically that the gradient in the structure of the TEM_02_ mode drives electron motion in the *Z* direction, subsequently producing a TEM_11_ mode in $|{\bar{E}}_{Z}|$. Projecting the Maxwell-Faraday equation along the *X*-axis, and using the properties of the Hermite polynomials (See the Supplementary Information file for further details), it can be shown that the *E*_*Z*_ field may be written:

1 $$\begin{array}{c}{E}_{Z} \sim 2{{\rm{TEM}}}_{11}-\frac{Z}{\sqrt{2}w(X)}[{{\rm{TEM}}}_{12}+{{\rm{TEM}}}_{10}],\\ \,\, \sim 2{{\rm{TEM}}}_{11}+{\mathscr{O}}(\frac{Z}{w(X)}).\end{array}$$

We note that the TEM_11_ is strictly due to the TEM_02_ mode, produced by the deceleration of the bipolar electron distribution. For a small $\frac{Z}{w(X)}$ ratio the *Z*-component of the electric field behaves as a TEM_11_ mode, in agreement with numerical simulation results.

The discussion thus far has focused on *ω*_*L*_ light. Transition radiation in the second harmonic is also generated and in high order modes, due to the fact that the overall electron bunch frequency (i.e. due to electrons at both poles) is 2*ω*_*L*_. In Fig. 5c, the spatial intensity profile of the 2*ω*_*L*_ light is plotted from the simulation with the predefined electron bunches, with arrows indicating the local polarisation direction. This demonstrates generation of a radially polarised mode.

Mode structures are also present at 2*ω*_*L*_ in the full laser-foil interaction (i.e. without the predefined plasma aperture). Figure 5d shows the scaling of the magnitude of the *E*_*Y*_ and *E*_*Z*_ fields at 2*ω*_*L*_, as a function of target thickness. For the thinnest target, *d* = 5 nm, the *E*_*Z*_ field is much weaker than the *E*_*Y*_ field, associated with the radiation emitted by the decelerating electron bunches. In this case, the spatial intensity distribution is dominated by the bipolar structure due to the electron bunches, as evidenced in Fig. 5e. As the target thickness increases to *d* = 20 nm, the magnitudes of *E*_*Y*_ and *E*_*Z*_ are approximately equal. Here, the *E*_*Y*_ field is in the form of a TEM_01_ mode, whilst the self-generated *E*_*Z*_ field has the structure of a TEM_10_ mode, at 2*ω*_*L*_. The superposition of these two modes, of equal amplitude, leads to the formation of a radially polarised mode. We also note that the radial mode generated in the full laser-foil simulation has a peak intensity of the order of the relativistic threshold. The fact that fundamental harmonic light can be filtered out, points to the possibility to develop this approach for the generation of relativistically intense (~3 × 10^18^ Wcm^−2^ in Fig. 5f) high order modes of 2*ω*_*L*_ light.

### Spatial mode variation of the 2*ω*_*L*_ light

**Figure 6:**
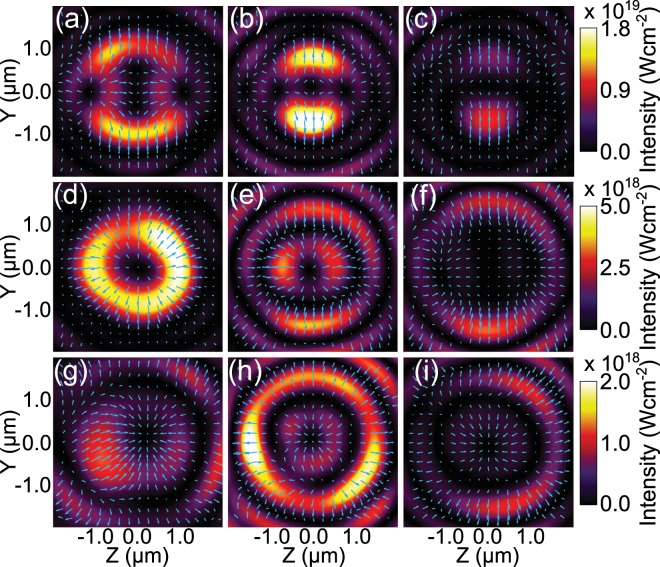
Spatial intensity distributions and electric field vectors (blue arrows) for 2*ω*_*L*_ light, from the full laser-foil simulations, measured 10 *μ*m behind the target rear. Three values of target thickness are compared; (**a**–**c**), 5 nm; (**d**–**f**), 20 nm; and (**g**–**i**), 30 nm. The spatial intensity distributions evolve temporally; panels (**a**,**d**,**g**) are compared 28 fs after the peak of the pulse, (**b**,**e**,**h**) are compared 40 fs after the peak of the pulse, and finally (**c**,**f**,**i)** are compared 52 fs after the peak of the pulse.

Next, we explore the extent to which the spatial mode varies with target thickness and the potential to tune this feature of the intense light generated. In Fig. 6, the evolution of the spatial intensity profile of the 2*ω*_*L*_ light is presented, for three target thicknesses, as follow: (a–c) *d* = 5 nm, (d–f) *d* = 20 nm and (g–i) *d* = 30 nm. The columns indicate different times, *t* = 28 fs, *t* = 40 fs and *t* = 52 fs (after the peak of the pulse) from left to right. *t* = 40 fs corresponds to the time at which the *d* = 20 nm target undergoes RSIT. In all cases, the distribution is determined at the same position in space (10 *μ*m behind the target rear), with the light averaged over a laser period.

The variation in spatial modes and polarisation with target thickness (i.e. comparing rows of Fig. 6) arises from the changing ratio of $|{\bar{E}}_{Y}|/|{\bar{E}}_{Z}|$, as discussed above. This points to the potential to tune this aspect of the intense light generated, by variation of target thickness or other parameters controlling the time at which RSIT occurs in the interaction. The mode structure is also observed to vary with time, when comparing the columns of Fig. 6, highlighting the potential to produce modes of intense laser light that dynamically vary on ultrafast timescales.

## Discussion

In summary, our results show that relativistically intense light in a TEM_00_ mode interacting with an ultrathin foil undergoing RSIT can generate higher order modes of intense light in the fundamental and second harmonic. We note that from an evaluation of the simulation results, the level of depolarisation of the generated and transmitted light is expected to be very small (see Supplementary Information file). The resultant light field and polarisation state vary with the degree of laser transmission. The work shows that measurement of these beam properties can thus be used to diagnose the intra-pulse time at which the plasma undergoes RSIT, which is a crucial parameter in optimising promising schemes for laser-driven ion acceleration^38^. Furthermore, the fact that they can be varied by choice of target thickness and that for certain thicknesses the mode evolves over the ultrashort duration of the interaction, opens up the possibility to develop this concept for ultrafast control of relativistically intense light fields. In future studies, we plan to explore the potential for this approach to add to the growing list of plasma optics and photonics schemes (e.g. for enhancing contrast^21–23^, amplification^17–19^, compression^20^ and polarisation control^26,27,40^) that are raising the potential for entirely plasma-based high fluence solutions to create extremely intense laser pulses^44^.

## Methods

### Experiment overview

The experiment was performed using the Gemini laser at the Rutherford Appleton Laboratory. Pulses of *p*-polarised, *λ*_*L*_ = 800 nm light (with 35 nm bandwidth), with energy equal to (3.1 ± 0.2) J (on-target) and pulse duration equal to (40 ± 5) fs (FWHM), were focused using an F/2 off-axis parabolic (OAP) mirror to a spot size of (3.9 ± 0.7) *μ*m in diameter (FWHM). The calculated peak intensity was (2.8 ± 0.4) × 10^20^ Wcm^−2^. A double plasma mirror configuration was used to enhance the intensity contrast to ~10^11^ and ~10^8^, at 1 ns and 2 ps, respectively, prior to the peak of the pulse. The targets were aluminium foils, with thickness *d*, varied in the range 16–60 nm and were irradiated at close to normal incidence.

**Figure 7:**
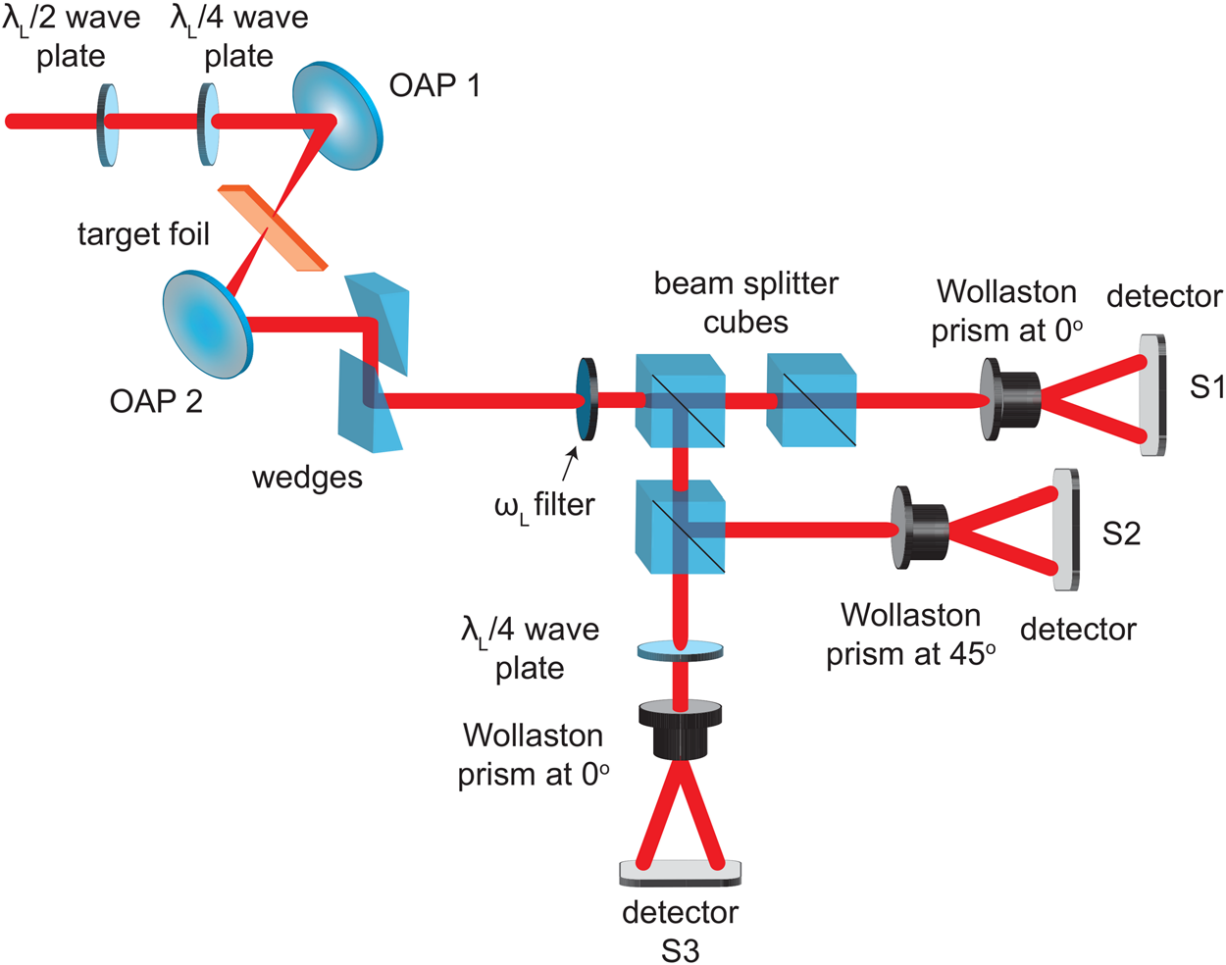
A schematic diagram of the experimental set-up detailing the polarisation diagnostic.

Figure 7 shows a schematic of the experimental set up for measuring the polarisation state of the light. The light which emerges at the rear of the target is collimated using a F/2 OAP (‘OAP 2’ in Fig. 7) and subsequently reflected off two wedged mirrors, reducing the energy such that it can pass to the external diagnostic (the Stokes polarimeter). It is first passed through an interference filter, transmitting light at the fundamental wavelength, *λ*_*L*_ = 800 nm, with a bandwidth of (40 ± 8) nm. It is then split into three paths by a series of non-polarising beam-splitter cubes, each with 50:50 transmission:reflection at *λ*_*L*_, enabling measurement of the corresponding Stokes parameters. A Wollaston prism is used in each of the paths to separate the light into two orthogonal polarisation states. The Wollaston angles were set to 0°, 45° and 0°, with respect to their fast axes, whilst the final path (along which the Wollaston angle was set to 0°) includes an additional *λ*_*L*_/4 wave plate after the Wollaston to separate the left and right handed circular polarisation states. The orthogonal polarisation states which emerge from the Wollaston prisms are in the form of two spots of light, which are recorded using an Andor Neo camera. These have a chip size of 16.6 × 14.0 mm, containing 2560 × 2160 pixels, and a dynamic range of 3 × 10^4^. This provides a time-integrated measurement of the polarisation state of the light.

### Stokes polarimetry

The Stokes representation of polarised light is employed in our analysis. The polarisation state of light is defined by a four-component Stokes vector. Each component in this vector is an experimentally measurable quantity, referred to as a Stokes parameter. The first parameter, *S*_0_, describes the total intensity of the light, whilst the remaining three components describe the difference in relative intensities of degenerate polarisation states. The term ‘degenerate states’ here refers to the six states commonly used in polarimetry, these are; linear polarisations at 0°, 90°, 45° and 135° (where the angle is defined as a rotation in the plane orthogonal to the direction of light propagation), and left and right handed circular polarisations. The Stokes parameters are defined as follows:

2 $${S}_{0}={I}_{0}+{I}_{90}=I=1$$

3 $${S}_{1}={I}_{0}-{I}_{90}=Ip\,\cos \,2\psi \,\cos \,2\chi $$

4 $${S}_{2}={I}_{45}-{I}_{135}=Ip\,\sin \,2\psi \,\cos \,2\chi $$

5 $${S}_{3}={I}_{RCP}-{I}_{LCP}=Ip\,\sin \,2\chi $$

where *I* is the total light intensity (here normalised to 1) and *I*_*n*_ is the intensity of light in the channel ‘*n*’ of the Stokes polarimeter. Here, *p* is the degree of polarisation, such that 0 ≤ *p* ≤ 1, and 2*ψ* and 2*χ* are characteristic angles. We assume that the light maintains its degree of polarisation, such that *p* = 1^46^. The Stokes parameters are then related as follows: $\sqrt{{S}_{1}^{2}+{S}_{2}^{2}+{S}_{3}^{2}}/{S}_{0}=1$.

The Stokes parameters may be visualised as co-ordinates in the so-called Poincaré sphere, in which the Stokes parameters form an orthonormal basis (as illustrated in the inset of Fig. 2b). In this interpretation, changes in the polarisation are seen as rotations of the Stokes vector, such that its tip moves to a different point on the surface of the Poincaré sphere. The Stokes parameters can then be combined to define an angle, called the angle of linear polarisation (AOLP), which is used to quantify the magnitude of the polarisation shifts which are measured experimentally. The AOLP is defined as follows^47^:

6 $${\rm{AOLP}}(^\circ )=\frac{1}{2}\arctan (\frac{{S}_{2}}{{S}_{1}})=\frac{1}{2}\arctan (\frac{{I}_{45}-{I}_{135}}{{I}_{0}-{I}_{90}})$$

For light that is completely polarised in the linear 0°–90° plane, i.e. the linear *p* and *s* states, the AOLP = 0°. For linear polarisation at an angle, the magnitude of the AOLP increases proportionally with the polarisation angle, up to a maximum value of 45°. This angle describes shifts of the linear polarisation state, and for completely polarised light is equal to the latitude in the Poincaré sphere.

The Stokes representation can also be used to describe the propagation of a polarisation state through a system of optical components. Each component is represented by a 4 × 4 matrix, a so-called Mueller matrix. A given optical system, such as the Stokes polarimeter, is then described by forming a composite matrix from the product of the Mueller matrices for each optical component. Whilst the Mueller matrices are readily obtained for commonly used equipment, such as polarisers and wave plates, this is not the case for many of the components employed in the Stokes polarimeter. We model the polarimeter by combining the Mueller matrices for the general effects of attenuation, rotation and retardation of the input polarisation vector. This leads to the formation of a system matrix, containing free parameters. These were fitted using a Monte-Carlo routine, which compares the output of the modelled system to known polarisation states, obtained during the initial calibration of the Stokes polarimeter.

### Simulations

The 3D simulations were performed using the fully relativistic particle-in-cell (PIC) code, EPOCH^43^. The simulation grid was composed of 1000 × 720 × 720 computational mesh cells, corresponding to a volume of 20 *μ*m × 20 *μ*m × 20 *μ*m. The laser enters this grid from the left boundary, with all other boundaries set to be free space. The pulse parameters were chosen to closely approximate those in the experiment; a Gaussian temporal profile with duration equal to 40 fs (FWHM), focused to a diameter of 3 *μ*m. The laser wavelength is *λ*_*L*_ = 800 nm and it is linearly polarised in the *Y* direction. The peak intensity was 6 × 10^20^ Wcm^−2^, such that the light level transmitted through the target approximately matched the experimental results. The time *t* = 0 fs is defined as when the peak of the laser pulse interacts with the center of the target.

The target comprised a layer of Al^13+^ ions, with *d* varied in the range 5–40 nm, with a 6 nm-thick layer of mixed C^6+^ and H^+^ ions on the surfaces to account for hydrocarbon contaminants in the experiment. To approximate the effect of the laser temporal-intensity contrast, the target ion and neutralising electron populations were pre-expanded to a Gaussian profile with a FWHM related to *d*, to achieve a maximum electron density of 30*n*_*c*_ (the areal density was kept equivalent to initially solid density aluminium; 444*n*_*c*_). The electron temperature was initially set to 100 keV, whilst the ions were set to 10 eV. This choice of electron temperature approximates the interaction of the expanding target plasma with the laser rising edge, yet ensures that the corresponding Debye length is resolved within the mesh size of the computational grid. The electron temperature and degree of pre-expansion are predicted based on plasma expansion estimates for the measured laser contrast, as described in Ref. ^45^. There were 22 particles per cell per species.

## Supplementary information

Supplementary Information.
