# Supplementary material for: High order mode structure of intense light fields generated via a laser-driven relativistic plasma aperture
Source: Sci Rep. 2020 Jan 9;10:105. doi: 10.1038/s41598-019-57119-x (PMC6952361; doi:10.1038/s41598-019-57119-x)
Supplement: Supplementary file 1 — Supplementary Information. [file 41598_2019_57119_MOESM1_ESM.pdf]

Supplementary Information: High order mode structure of intense light fields generated via a laser-driven relativistic plasma aperture

M. J. Duff[1], R. Wilson[1], M. King[1], B. Gonzalez-Izquierdo[1], A. Higginson[1], S. D. R. Williamson[1], Z. E. Davidson[1], R. Capdessus[1], N. Booth[2], S. Hawkes[2], D. Neely[1,2], R. J. Gray[1], and P. McKenna[1,*]

[1]SUPA Department of Physics, University of Strathclyde, Glasgow, G4 0NG, UK
[2]Central Laser Facility, STFC Rutherford Appleton Laboratory, Oxfordshire, OX11 0QX, UK
[*]paul.mckenna@strath.ac.uk

Calibration of the Stokes polarimeter

Calibration of the Stokes polarimeter was performed by varying the polarisation via a combination of a quarter ($\lambda_L/4$) and a half ($\lambda_L/2$) wave plate positioned in the beam path, such that any elliptical polarisation state could be generated. There were no targets in place for this calibration, which was performed using the Gemini laser in low power (continuous wave) mode. Fixing one of the wave plates, whilst the other was rotated in 5° increments, in the range 0°-180°, the brightness of the spots on each of the three polarimeter cameras was measured for each polarisation state. This process was then repeated, swapping the fixed and rotating wave plates. This data was used in a Monte-Carlo routine to fit the free parameters in the system matrix.

For full power shots with targets in place, the integrated counts within each of the spots is measured, enabling the relative brightness of the spots on each camera to be determined. To make this measurement, the center of each spot is located, then the total number of counts within a circular area which encloses the entire spot is recorded. The radius of the circle is increased in five increments, and the relative brightness at each radius is determined by comparing with the number of counts in the second spot, at the corresponding radius. The relative brightness of the spots are then compared to the set of possible outputs from the Stokes polarimeter, enabling the full Stokes parameters and thus the final polarisation state to be determined. In general, there is a set of final states which explain the experimental data, within the level of uncertainty in the measurement of the relative brightness of the spots. If this is the case, the Stokes parameters are averaged over all possible final states.

Frequency resolved optical gating (FROG) measurements

To measure the temporal-intensity profile of the light detected at the rear of the target, a fraction of the collimated beam, after the second F/2 parabola and before the wedged mirrors, was sampled using an elliptical pick-off mirror. This light was directed out of the target chamber through a thin fused silica window, into a frequency resolved optical gating (FROG) diagnostic. The particular type of FROG employed was the GRENOUILLE, described in detail in Supplementary Ref.[1]. This enabled the temporal-intensity profile and the instantaneous frequency of the light detected at the target rear, to be determined. The light entering the FROG was filtered using an (800±35) nm interference filter, and attenuated using a neutral density filter.

During full power shots, an image is obtained using the FROG (a so-called trace), with axes corresponding to time (i.e. the delay between two separate pulses, obtained by passing the incident light through a Fresnel biprism) and angular frequency. To recover the temporal intensity profile of the light, it was necessary to employ the FROG pulse-retrieval software. This is because there is no function which enables the temporal intensity profile to calculated directly from the experimentally obtained FROG trace. Instead, the pulse-retrieval software makes an initial estimate of the temporal profile of the electric field and calculates the corresponding FROG trace. The initial form of the electric field is iteratively improved until good agreement is achieved between the experimentally obtained trace and that calculated using the software. In order to determine the direction of time (and therefore whether a pulse is positively or negatively chirped), the FROG must first be calibrated. This was achieved via a series of calibration shots with no target in place. The pulse duration was stretched by adding positive chirp, via an opto-acoustic device known as a Dazzler. The presence of chirp leads to a gradient in the FROG trace; given that it is known whether the chirp is positive or negative, it is then possible to determine the direction of time. In addition, varying the pulse energy enabled the magnitude of non-linear optical effects to be determined. The FROG traces were corrected for such effects, which add group velocity dispersion to the propagating light.

Evaluating potential depolarisation

We cannot assess the extent to which the generated and transmitted light may be depolarised (i.e. with polarisation states randomly distributed) in the experiment, but we can explore this with the simulation results. We do this by evaluating the

distribution of the angle $\theta = \arctan(E_Z/E_Y)$ between the individual spatial E_Y and E_Z electric field components from the 3D simulations, over a spatial extent X=6 μm to X=12 μm at t=40 fs for the d=10 nm and d=30 nm targets. The resultant magnitude of these $|E|$ components is used to weight the contribution of each angle and the distribution is normalised to the maximum total $|E|$ (explicitly, $|E| = \sqrt{E_Y^2 + E_Z^2}$). This is shown in Figure 1a and 1b for the two target thicknesses. A perfectly linearly polarised pulse in the Y direction will have a single value at $\pm\pi/2$. For the light transmitted through the d=10 nm target, there is a small degree of broadening induced by the generated light polarised in the Z direction. For the d=30 nm case, however, the distribution is broader, due to an increased ratio of total $|E_Z|$ to $|E_Y|$, with an increase in the base noise level. This noise level arises due to a combination of numerical noise in the simulation and the degree of depolarisation of the light.

The results are compared to a theoretical model used to evaluate the superposition of a simple Gaussian TEM_{00} beam polarised in the Y direction and a TEM_{11} beam polarised in the Z direction, i.e. without depolarisation. A full 3D spatial grid identical to the sampled spatial extent of the simulations was used. The ratio of total $|E_Z|$ and $|E_Y|$ determined from the same spatial extent in the simulations was used to reduce the total $|E_Z|$ in the theoretical model for both the d=10 nm and d=30 nm targets. The beam waist used in each model was also determined from the averaged spatial profile of the beam across the sampled spatial extent in each simulation.

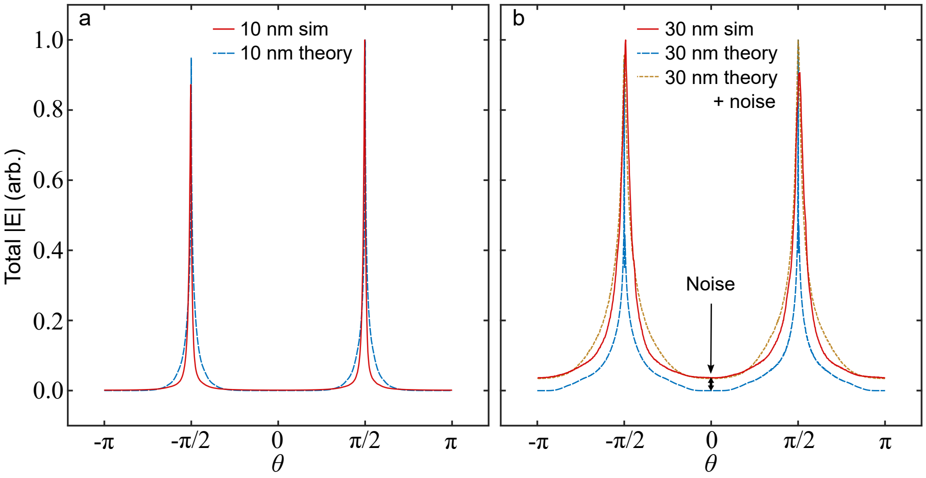

Figure 1. Angular distribution of electric field components E_Y and E_Z weighted by the resultant magnitude, in the simulation (red) and theoretical model for the case of no depolarisation (blue), for: **a**, d=10 nm; and **b**, d=30 nm. The thicker target case includes the addition of noise to the theoretical model (orange). The results are sampled over a spatial extent X=6 μm to X=12 μm at t=40 fs and are normalised to each maximum total $|E|$ in all cases.

In Figure 1a, we see good agreement between the electric field angular distributions extracted from the simulation results and that expected from the theoretical model, for d=10 nm. The slight difference in width could be due to variation in the temporal profile and/or pulse separation which was not included in the model. For the d=30 nm case, shown in Figure 1b, the simulation results also follow the model predictions, but with an off-set caused by an increase in the base level of noise. As noted above, this includes numerical noise within the simulation, which is relatively higher in thicker targets because the total detected light signal is lower, and so the contribution due to depolarisation of the light cannot easily be deconvolved and quantified. Nevertheless, the fact that the electric field distributions in the simulations are in overall very good agreement with that expected from the model indicates that very little depolarisation takes place, with negligible amounts for the thinner targets.

Further details on the analytical model for the generation of the TEM_{11} mode

The complex field amplitude of the $TEM_{n,m}$ (or Hermite-Gauss) mode is given in Cartesian coordinates by:

$$E_{n,m}(Y,Z) = E_0 \left(\frac{w_0}{w(X)}\right) H_n \left(\frac{\sqrt{2}Y}{w(X)}\right) H_m \left(\frac{\sqrt{2}Z}{w(X)}\right) \times \exp\left[-\frac{r^2}{w^2(X)} - ik_L X - ik_L \left(\frac{r^2}{2R(X)}\right) + i(m+n+1)\xi(X)\right] \quad (1)$$

where $r^2 = Y^2 + Z^2$, $w(X)$ and $R(X)$ are the waist size and the radius of curvature, ξ is the Guoy phase and H_m denotes the mth Hermite polynomial. The transmitted light is a combination of a TEM_{00} mode and a TEM_{02} mode, produced by the deceleration

of the bipolar electron distribution, polarised along the Y axis. The absolute value of the field (E_Y) may be written:

$$E_Y = E_0 \left(\frac{w_0}{w(X)}\right) \exp\left(-\frac{r^2}{w^2(X)}\right) \left[H_2\left(\frac{\sqrt{2}Z}{w(X)}\right) + 1\right]. \tag{2}$$

Projecting the Maxwell-Faraday equation along the X axis yields:

$$\begin{aligned} \partial_Y E_Z &= \partial_Z E_Y, \\ \Rightarrow E_Z &= \int \partial_Z E_Y dY. \end{aligned} \tag{3}$$

Using the properties of the Hermite polynomials, $\partial_u H_n(u) = 2nH_{n-1}(u)$, we can show that

$$\begin{aligned} \partial_Z E_Y = \quad & E_0 \left(\frac{w_0}{w(X)}\right) \exp\left(-\frac{r^2}{w^2(X)}\right) \\ & \times \left\{\frac{4\sqrt{2}}{w(X)} H_1\left(\frac{\sqrt{2}Z}{w(X)}\right) - \frac{2Z}{w^2(X)}\left[H_2\left(\frac{\sqrt{2}Z}{w(X)}\right) + 1\right]\right\}, \end{aligned} \tag{4}$$

hence,

$$\begin{aligned} E_Z = \quad & E_0 \left(\frac{w_0}{w(X)}\right) \times \frac{\sqrt{\pi}}{2} w(X) \operatorname{erf}\left(\frac{Y}{w(X)}\right) \exp\left(-\frac{Z^2}{w^2(X)}\right) \\ & \times \left\{\frac{4\sqrt{2}}{w(X)} H_1\left(\frac{\sqrt{2}Z}{w(X)}\right) - \frac{2Z}{w^2(X)}\left[H_2\left(\frac{\sqrt{2}Z}{w(X)}\right) + 1\right]\right\} \end{aligned} \tag{5}$$

Using the error function's MacLaurin series, such that $\operatorname{erf}(u) \sim \frac{2u\exp(-u^2)}{\sqrt{\pi}}$, we finally obtain

$$\begin{aligned} E_Z \sim \quad & E_0 \left(\frac{w_0}{w(X)}\right) H_1\left(\frac{\sqrt{2}Y}{w(X)}\right) \exp\left(-\frac{r^2}{w^2(X)}\right) \\ & \times \left\{2H_1\left(\frac{\sqrt{2}Z}{w(X)}\right) - \frac{Z}{\sqrt{2}w(X)}\left[H_2\left(\frac{\sqrt{2}Z}{w(X)}\right) + 1\right]\right\}. \end{aligned} \tag{6}$$

where $H_1(u) = 2u$ has been used. Using the definition in Supplementary Eqn. (1) the electric field E_Z may be expressed as

$$\begin{aligned} E_Z \sim \quad & 2\mathrm{TEM}_{11} - \frac{Z}{\sqrt{2}w(X)}\left[\mathrm{TEM}_{12} + \mathrm{TEM}_{10}\right], \\ \sim \quad & 2\mathrm{TEM}_{11} + \mathscr{O}\left(\frac{Z}{w(X)}\right). \end{aligned} \tag{7}$$

We note that the TEM_{11} is strictly due to the TEM_{02} mode, produced by the deceleration of the bipolar electron distribution. For a small $\frac{Z}{w(X)}$ ratio the Z-component of the electric field behaves as a TEM_{11} mode, in agreement with numerical simulation results.

References

1. Trebino, R. *Frequency-Resolved Optical Gating: The Measurement of Ultrashort Laser Pulses* (Kluwer Academic Publishers, Boston, 2002).
